# A minimally invasive dried blood spot biomarker test for the detection of Alzheimer’s disease pathology

**DOI:** 10.1038/s41591-025-04080-0

**Published:** 2026-01-05

**Authors:** Hanna Huber, Laia Montoliu-Gaya, Wagner S. Brum, Jakub Vávra, Yara Yakoub, Haley Weninger, Luisa Sophie Braun-Wohlfahrt, Joel Simrén, Mercé Boada, Agustín Ruiz, Amanda Cano, Adelina Orellana, Sergi Valero, Laia Cañada, Natalia Tantinya, Ana Belen Nogales, Pilar Sanz-Cartagena, Anna Dittrich, Ingmar Skoog, Millie Sander-Long, Clive Ballard, Megan Richards, Mary O’Leary, Frederikke Kragh Clemmensen, Hannah H. D. Wandall, Daniele Altomare, Valentina Cantoni, Erik Stomrud, Sebastian Palmqvist, Alberto Lleo, Daniel Alcolea, Maria Carmona Iragui, Aida Sanjuan Hernandez, Bessy Benejam, Laura Videla Toro, Alpana Singh, Marisa N. Denkinger, Anja Hviid Simonsen, Silke Kern, Anne Corbett, Juan Fortea, Lee Honigberg, Barbara Borroni, Oskar Hansson, Xavier Morató, Kaj Blennow, Henrik Zetterberg, Nicholas J. Ashton

**Affiliations:** 1 Department of Psychiatry and Neurochemistry, Institute of Neuroscience and Physiology, The Sahlgrenska Academy at the University of Gothenburg, Mölndal, Sweden; 2 German Center for Neurodegenerative Diseases, Bonn, Germany; 3 University Hospital Bonn, Department of Old Age Psychiatry and Cognitive Disorders, Bonn, Germany; 4 Graduate Program in Biological Sciences: Biochemistry, Universidade Federal do Rio Grande do Sul (UFRGS), Porto Alegre, Brazil; 5 Integrated Program in Neuroscience, McGill University, Montreal, Quebec Canada; 6 Research Center of the Douglas Mental Health University Institute, Montreal, Quebec Canada; 7 Ace Alzheimer Center Barcelona-Universitat Internacional de Catalunya, Barcelona, Spain; 8 Networking Research Center on Neurodegenerative Diseases (CIBERNED), Instituto de Salud Carlos III, Madrid, Spain; 9 Region Västra Götaland, Sahlgrenska University Hospital, Department of Neuropsychiatry, Mölndal, Sweden; 10 College of Medicine and Health, University of Exeter, Exeter, UK; 11 Danish Dementia Research Centre, Department of Neurology, Copenhagen University Hospital - Rigshospitalet, Copenhagen, Denmark; 12 Department of Clinical and Experimental Sciences, University of Brescia, Brescia, Italy; 13 Competence Centre on Ageing (CCA), Department of Business Economics, Health and Social Care (DEASS), University of Applied Sciences and Arts of Southern Switzerland (SUPSI), Manno, Switzerland; 14 Clinical Memory Research Unit, Department of Clinical Sciences Malmö, Lund University, Lund, Sweden; 15 Memory Clinic, Skåne University Hospital, Malmö, Sweden; 16 Sant Pau Memory Unit, Department of Neurology, Biomedical Research Institute Sant Pau, Hospital de la Santa Creu i Sant Pau, Barcelona, Spain; 17 Centro de Investigación Biomédica en Red de Enfermedades Neurodegenerativas. CIBERNED, Barcelona, Spain; 18 Barcelona Down Medical Center, Fundació Catalana Síndrome de Down, Barcelona, Spain; 19 Banner Sun Health Research Institute, Sun City, AZ USA; 20 Neuropsychiatric Epidemiology Unit, Department of Psychiatry and Neurochemistry, Institute of Neuroscience and Physiology, Sahlgrenska Academy, Centre for Ageing and Health (AGECAP) at the University of Gothenburg, Mölndal, Sweden; 21 ALZpath, Inc., Carlsbad, CA USA; 22 Molecular Markers Laboratory, IRCCS Istituto Centro San Giovanni di Dio Fatebenefratelli, Brescia, Italy; 23 Clinical Neurochemistry Lab, Sahlgrenska University Hospital, Mölndal, Sweden; 24 Paris Brain Institute, ICM, Pitié-Salpêtrière Hospital, Sorbonne University, Paris, France; 25 Neurodegenerative Disorder Research Center, Division of Life Sciences and Medicine, and Department of Neurology, Institute on Aging and Brain Disorders, University of Science and Technology of China and First Affiliated Hospital of USTC, Hefei, China; 26 Department of Neurodegenerative Disease, UCL Institute of Neurology, London, UK; 27 UK Dementia Research Institute at UCL, London, UK; 28 Hong Kong Center for Neurodegenerative Diseases, InnoHK, Hong Kong, China; 29 Wisconsin Alzheimer’s Disease Research Center, University of Wisconsin School of Medicine and Public Health, University of Wisconsin-Madison, Madison, WI USA; 30 Centre for Brain Research, Indian Institute of Science, Bangalore, India; 31 Banner Alzheimer’s Institute and University of Arizona, Phoenix, AZ USA

**Keywords:** Diagnostic markers, Alzheimer's disease

## Abstract

Blood biomarkers have emerged as accurate tools for detecting Alzheimer’s disease (AD) pathology, offering a minimally invasive alternative to traditional diagnostic methods such as imaging and cerebrospinal fluid (CSF) analysis. Yet, the logistics surrounding venipuncture for blood collection, although considerably simpler than the acquisition of imaging and CSF, require precise processing and storage specific to AD biomarkers that are still guided by medical personnel. Consequently, limitations in their widescale use in research and broader clinical implementation exist. The DROP-AD project investigates the potential of dried plasma spot (DPS) and dried blood spot (DBS) analysis, derived from capillary blood, for detecting AD biomarkers, including phosphorylated tau at amino acid 217 (p-tau217), glial fibrillary acidic protein and neurofilament light. Here, 337 participants from 7 centers were included, with 304 participants providing paired capillary DPS or DBS and venous plasma samples. We observed strong correlations between DPS p-tau217 and venous plasma p-tau217 (*r*_S_ = 0.74, *P* < 0.001). DPS p-tau217 progressively increased with increasing disease severity, and showed good accuracy in predicting CSF biomarker positivity (area under the curve = 0.864). Similarly, we demonstrated the successful detection of glial fibrillary acidic protein and neurofilament light with strong correlations between DBS and DPS, respectively, using paired venous plasma samples. Notably, the method was also effective in individuals with Down syndrome, a population at high genetic risk for AD but in whom standard blood sampling by venipuncture may be more complicated, revealing elevated biomarkers in those with dementia compared with asymptomatic individuals. The study also explored unsupervised blood collection, finding high concordance between supervised and self-collected samples. These findings underscore the potential of dried blood collection and capillary blood as a minimally invasive, scalable approach for AD biomarker testing in research settings. Yet, further refinement of collection and analytical protocols is needed to fully translate this approach to be viable and useful as a clinical tool.

## Abstract (web summary)

This multicenter study demonstrates use of dried and capillary blood as a minimally invasive, scalable approach for Alzheimer’s biomarker testing in research, with potential as a widely scalable population-based research approach, especially in resource-limited settings.

## Main

In less than a decade, the development of blood biomarkers for the identification of AD pathology has transitioned from a promising research endeavor to a valued tool that is now included in research diagnostic criteria^1^ and is increasingly being adopted in clinical practice. Phosphorylated tau at amino acid 217 (p-tau217) has emerged as an early and accurate AD blood biomarker^2,3^, offering higher accuracy compared with other putative blood biomarkers for detecting cerebral amyloid-β (Aβ) pathology^4^, a required hallmark for an AD diagnosis^5,6^. Several blood p-tau217 assays, spanning different immunological detection methods and mass spectrometry techniques^7^ are now available with some—but not all—meeting the recommended criteria for clinical usefulness, approved for clinical use, or currently under regulatory evaluation^8^. Thus, a cost-effective and timely tool is now available to identify individuals who may benefit from approved and emerging treatments or to monitor disease progression^9^. Specifically, the most likely clinical applications of blood p-tau217 will be based on a two-cutoff approach^10^ aimed at identifying people at either very high or very low risk of brain amyloidosis and for whom additional biomarker investigations are unnecessary, thereby lowering the need for positron emission tomography (PET) or CSF testing^11^. Other supportive blood biomarkers also offer insights into disease pathophysiology. Specifically, glial fibrillary acidic protein (GFAP), a marker of astrogliosis, has been associated with the onset of Aβ deposition^12,13^; and neurofilament light (NfL), a marker for axonal degeneration across neurodegenerative diseases^14^, has also been developed and is widely deployed in research and some clinical and therapeutic settings.

Although current guidelines recommend AD blood biomarker testing for symptomatic individuals^15^, there is also the potential to screen cognitively unimpaired (CU) older adults using a simplified test in a research setting and prevention strategies. This is because of the expectation of improved effectiveness of disease-modifying therapies targeting amyloid pathology in earlier disease stages, with trials currently ongoing^16^. Moreover, broader implementation of blood biomarkers will likely substantially advance the treatment, management and biological understanding of AD and related disorders in populations and communities currently underrepresented in research; for example, in individuals with Down syndrome (DS).

Substantial efforts have been made to ensure that blood tests become widely accessible, rather than confined to specialized laboratories. A major advancement in this area is the development of high-performing commercial and fully automated immunoassays for AD blood biomarkers, in particular p-tau217^17^. These fully automated immunoassays demonstrate performance identical or almost identical to immunoprecipitation mass spectrometry, as shown in a series of papers^17–21^. Although immunoprecipitation mass spectrometry remains difficult for widespread research and clinical implementation because of its high costs and limited instrument availability, automated immunoassays offer reliable and scalable solutions that address these limitations. Yet, for their global adoption to be fully realized, logistical challenges surrounding blood collection—such as the need for timely and standardized sample handling and storage^22^, as well as limited access to phlebotomy services—must be overcome to avoid constraining the impact of blood-based biomarker testing.

The DROP-AD project aims to streamline blood sample collection for larger-scale research, therapeutic trial enrollment and, potentially, clinical care, by introducing an alternative method that addresses the logistical challenges of traditional venipuncture blood collection and processing. By using capillary blood collected on DBS or DPS cards, the latter needed for p-tau217, reliance on guided venipuncture, immediate centrifugation and temperature-controlled shipment is eliminated. This approach enables a simplified, and potentially self-administered, protocol using fingertip blood collection. We have previously demonstrated the feasibility of measuring AD biomarkers from dried blood spots^23^, with the blood source being venous, followed by manual transfer onto a card for shipping and storage advantages. Here we extend this previous work by evaluating the feasibility of remote biomarker assessment using capillary blood, obtained by fingerstick collection, thus potentially self-collected and remote. A total of 337 participants were recruited across 7 European centers to assess the quantification of key AD pathology and neurodegeneration biomarkers—plasma p-tau217, NfL and GFAP—from capillary-derived blood collected from the finger. These values were directly compared with those obtained by standard venous plasma sampling, as well as to CSF biomarker concentrations routinely used in clinical diagnostics.

## Results

**Fig. 1:**
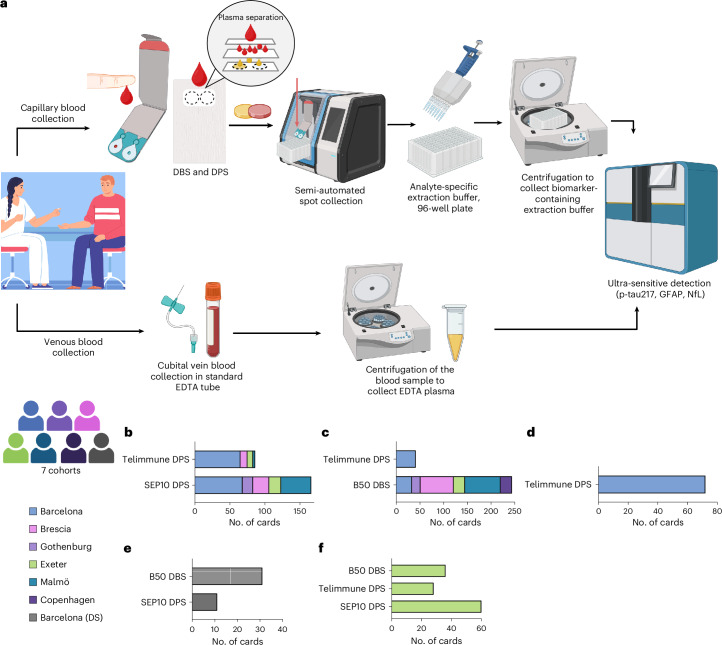
DROP-AD in-house collection and extraction protocol and testing procedures. **a**, Collection and processing of venous plasma and capillary DPS and DBS samples. For DPS and DBS sample collection, a finger prick was carried out by trained study personnel and a few drops of capillary blood were spotted onto DPS and DBS collection devices. DPS and DBS were collected via semi-automated spot collectors and incubated with analyte-specific extraction buffer in a 96-well filter plate. After incubation and centrifugation, the eluate was immediately measured using ultrasensitive immunoassays on the single molecule array platform. **b**–**f**, Participant numbers and collection device numbers per cohort: capillary p-tau217 (**b**), capillary GFAP (**c**), capillary NfL (**d**), DS cohort (**e**) and self-sampling cohort (**f**). Panel **a** created using BioRender.com. Source data

We recruited 337 participants (mean (s.d.) age, 70.8 (11.7) years; 167 women (53.4%) (Table 1) from 7 centers. Depending on the research site, and the evolution of the DROP-AD project, participants followed different capillary blood collection and testing procedures, which are summarized in Fig. 1. Cohort characteristics are shown in Supplementary Table 1.

**Table 1:** Participant characteristics

		Barcelona (*n* = 133)	Barcelona (DS) (*n* = 31)	Brescia (*n* = 33)	Copenhagen (*n* = 24)	Exeter (*n* = 25)	Gothenburg (*n* = 15)	Malmö (*n* = 76)	Total (*n* = 337)
Age	Mean (s.d.)	75.0 (7.73)	47.6 (8.68)	65.7 (8.97)	72.3 (6.27)	65.2 (6.10)	68.1 (6.70)	77.3 (8.10)	70.9 (11.4)
	Median (Q1–Q3)	77.0 (72.8–80.0)	48.0 (43.0–53.0)	67.5 (60.0–72.5)	73.9 (67.6–76.3)	64.0 (62.0–69.0)	69.0 (65.5–72.5)	79.3 (73.7–83.3)	74.0 (64.0–79.0)
	Missing	1 (0.8%)	1 (3.2%)	1 (3.0%)	1 (4.2%)	0 (0%)	1 (6.7%)	0 (0%)	5 (1.5%)
Sex	Female	50 (37.6%)	20 (64.5%)	15 (45.5%)	16 (66.7%)	10 (40.0%)	7 (46.7%)	41 (53.9%)	159 (47.2%)
	Male	82 (61.7%)	11 (35.5%)	17 (51.5%)	8 (33.3%)	15 (60.0%)	7 (46.7%)	35 (46.1%)	175 (51.9%)
	Missing	1 (0.8%)	0 (0%)	1 (3.0%)	0 (0%)	0 (0%)	1 (6.7%)	0 (0%)	3 (0.9%)
MMSE	Mean (s.d.)	24.8 (4.26)	NA	25.7 (7.46)	25.0 (4.89)	NA	26.1 (3.83)	26.5 (2.86)	25.3 (4.65)
	Median (Q1–Q3)	26.0 (22.0–28.0)	NA	30.0 (23.0–30.0)	26.0 (23.5–29.0)	NA	27.0 (24.3–29.0)	27.0 (25.0–28.3)	26.0 (23.0–29.0)
	Missing	3 (2.3%)	31 (100%)	1 (3.0%)	0 (0%)	25 (100%)	1 (6.7%)	32 (42.1%)	93 (27.6%)
Clinical diagnosis	CU	4 (3.0%)	0 (0%)	18 (54.5%)	0 (0%)	0 (0%)	0 (0%)	18 (23.7%)	40 (11.9%)
	MCI	83 (62.4%)	0 (0%)	0 (0%)	0 (0%)	0 (0%)	0 (0%)	10 (13.2%)	93 (27.6%)
	AD	31 (23.3%)	0 (0%)	6 (18.2%)	0 (0%)	0 (0%)	0 (0%)	12 (15.8%)	49 (14.5%)
	Non-AD	10 (7.5%)	0 (0%)	8 (24.2%)	0 (0%)	0 (0%)	0 (0%)	19 (25.0%)	37 (11.0%)
	aDS	0 (0%)	18 (58.1%)	0 (0%)	0 (0%)	0 (0%)	0 (0%)	0 (0%)	18 (5.3%)
	pDS	0 (0%)	2 (6.5%)	0 (0%)	0 (0%)	0 (0%)	0 (0%)	0 (0%)	2 (0.6%)
	dDS	0 (0%)	9 (29.0%)	0 (0%)	0 (0%)	0 (0%)	0 (0%)	0 (0%)	9 (2.7%)
	Missing	5 (3.8%)	2 (6.5%)	1 (3.0%)	24 (100%)	25 (100%)	15 (100%)	17 (22.4%)	89 (26.4%)
AD pathology (CSF p-tau181/Aβ42)	Negative	51 (38.3%)	0 (0%)	3 (9.1%)	9 (37.5%)	0 (0%)	7 (46.7%)	25 (32.9%)	95 (28.2%)
	Positive	80 (60.2%)	0 (0%)	4 (12.1%)	14 (58.3%)	0 (0%)	6 (40.0%)	15 (19.7%)	119 (35.3%)
	Missing	2 (1.5%)	31 (100%)	26 (78.8%)	1 (4.2%)	25 (100%)	2 (13.3%)	36 (47.4%)	123 (36.5%)
AD pathology (CSF Aβ42/Aβ40)	Negative	38 (28.6%)	0 (0%)	1 (3.0%)	3 (12.5%)	0 (0%)	4 (26.7%)	17 (22.4%)	63 (18.7%)
	Positive	93 (69.9%)	0 (0%)	2 (6.1%)	20 (83.3%)	0 (0%)	10 (66.7%)	23 (30.3%)	148 (43.9%)
	Missing	2 (1.5%)	31 (100%)	30 (90.9%)	1 (4.2%)	25 (100%)	1 (6.7%)	36 (47.4%)	126 (37.4%)
Capillary p-tau217	Mean (s.d.)	0.024 (0.025)	0.013 (0.014)	0.017 (0.014)	NA	0.015 (0.013)	0.020 (0.022)	0.012 (0.008)	0.020 (0.022)
	Median (Q1–Q3)	0.016 (0.009–0.028)	0.010 (0–0.020)	0.015 (0.006–0.024)	NA	0.012 (0.006–0.019)	0.011 (0.006–0.023)	0.010 (0.006–0.015)	0.013 (0.007–0.026)
	Missing	0 (0%)	22 (71.0%)	0 (0%)	24 (100%)	0 (0%)	0 (0%)	24 (31.6%)	70 (20.8%)
Capillary GFAP	Mean (s.d.)	NA	10.4 (12.3)	NA	7.60 (4.22)	11.4 (3.88)	7.80 (1.70)	8.81 (5.19)	9.25 (7.04)
	Median (Q1–Q3)	NA	6.70 (4.10–10.1)	NA	6.88 (5.04–8.40)	12.6 (7.69–14.4)	7.80 (7.20–8.40)	8.04 (4.82–11.5)	7.62 (4.99–11.5)
	Missing	133 (100%)	0 (0%)	33 (100%)	0 (0%)	7 (28.0%)	13 (86.7%)	1 (1.3%)	187 (55.5%)
Capillary NfL	Mean (s.d.)	NA	NA	NA	NA	NA	NA	NA	NA
	Median (Q1–Q3)	NA	NA	NA	NA	NA	NA	NA	NA
	Missing	133 (100%)	31 (100%)	33 (100%)	24 (100%)	25 (100%)	15 (100%)	76 (100%)	337 (100%)

NA, not available; Q, quartile.

### Correlation of capillary DPS p-tau217 with venous plasma p-tau217

**Fig. 2:**
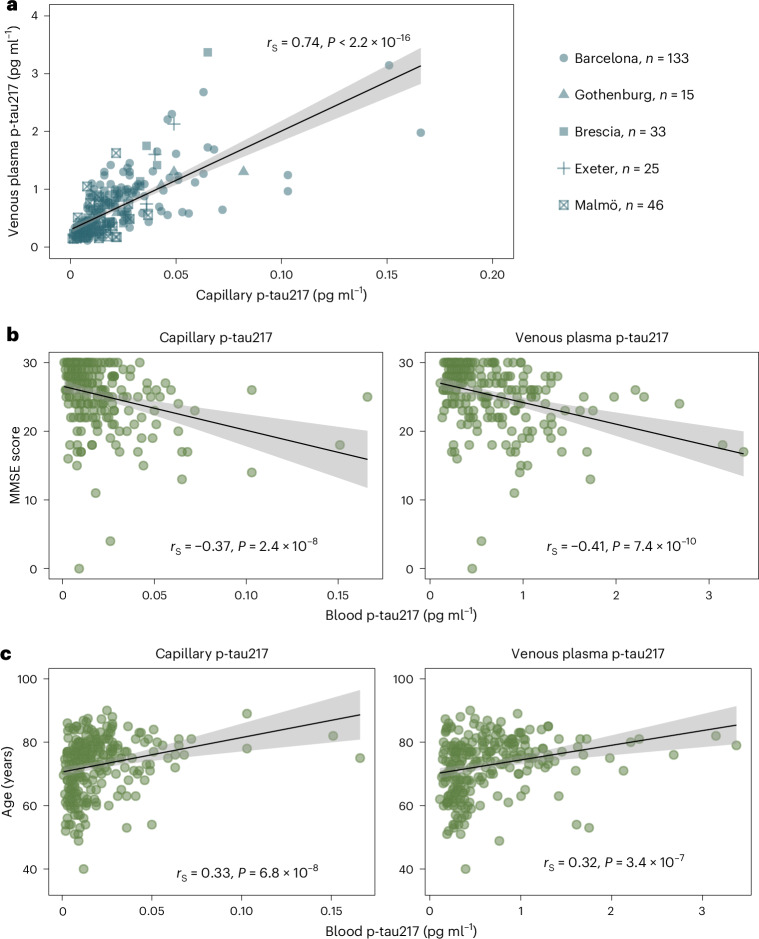
Correlations between capillary p-tau217 with venous plasma p-tau217, cognition and age. **a**, Correlation between capillary p-tau217 and venous plasma p-tau217 (*n* = 252). Dots correspond to individual data points. **b**, Correlation between capillary p-tau217 and MMSE (*n* = 209). Left: capillary p-tau217. Right: venous plasma p-tau217. **c**, Correlation between capillary p-tau217 and age (*n* = 249). Left: capillary p-tau217. Right: venous plasma p-tau217. A mean regression line is presented in all panels, with ribbons representing 95% CI. For numerical representation of the correlation, we present Spearman coefficients alongside their *P* values. Statistical tests were two-sided. Source data

**Extended Data Fig. 1:**
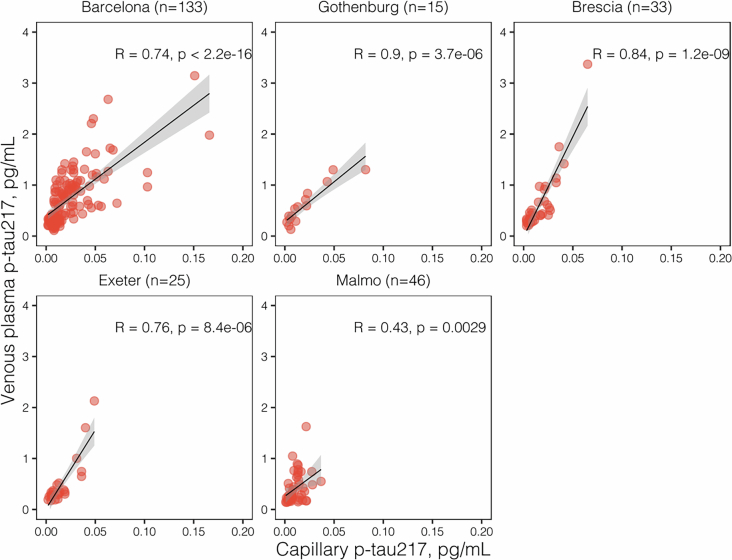
Correlation between capillary p-tau217 and venous plasma p-tau217 by cohort. Scatterplots display the relationship between venous plasma p-tau217 (y-axis) and capillary p-tau217 (x-axis), stratified in each panel according to the cohort in which biomarkers were measured. Spearman correlation coefficients are displayed alongside their p-values and a mean regression line with 95% confidence intervals. Of note, all venous plasma and capillary p-tau217 measurements were performed with the ALZpath assay, with the Malmö cohort being the exception, where the venous plasma values were acquired with the Lilly MSD assay, hence different correlation magnitudes. Number of individuals in each plot is presented in the figure. Statistical tests were two-sided. P-tau 217 = Phosphorylated tau 217. Source data

**Extended Data Fig. 2:**
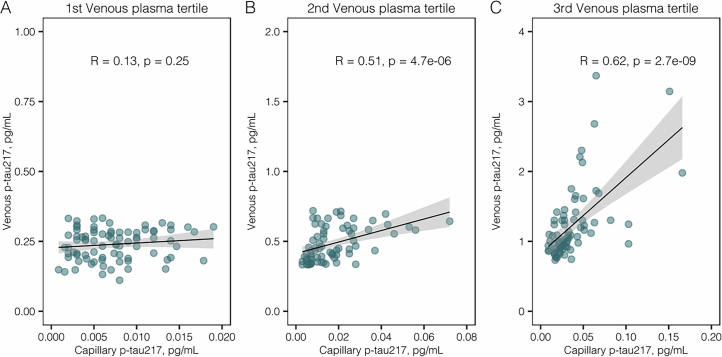
The relationship between capillary and venous plasma p-tau217 concentrations by plasma tertile. Scatterplots representing the association between venous plasma (y-axis) and capillary (x-axis) p-tau217 values, stratified by tertiles (n = 84 for each panel; **A**, in the first venous plasma tertile; **B**, in the second venous plasma tertile; and **C**, in the third venous plasma tertile) of plasma p-tau217 distribution so as to visualize the magnitude of venous plasma-capillary correlation across different concentration strata. Spearman correlation coefficients are shown alongside their p-values and a mean regression line with 95% confidence intervals. Statistical tests were two-sided. P-tau 217 = Phosphorylated tau 217. Source data

In total, 252 participants (mean (s.d.) age, 72.7 (9.0) years; 143 women (56.7%)) provided paired capillary DPS samples and venous plasma samples. We found a high correlation between DPS p-tau217 and venous plasma p-tau217 across all merged cohorts (Spearman’s rank correlation (*r*_S_) = 0.74, 95% confidence interval (CI) 0.678–0.791; *P* < 0.001) (Fig. 2a). The strength of this correlation varied among participating centers (Extended Data Fig. 1) and was highest in the Gothenburg cohort (*r*_S_ = 0.904, 95% CI 0.731–0.968; *P* < 0.0001) and the Brescia cohort (*r*_S_ = 0.838, 95% CI 0.694–0.917; *P* < 0.0001), followed by the Exeter (*r*_S_ = 0.765, 95% CI 0.530–0.891; *P* < 0.0001) and Barcelona (*r*_S_ = 0.735, 95% CI 0.646–0.805; *P* < 0.0001) cohorts, and the Malmö cohort (*r*_S_ = 0.429, 95% CI 0.159–0.640; *P* < 0.001), the only cohort in which a different assay (Lilly^2^) for venous plasma was used. To further demonstrate the strength the relationship between capillary and venous blood, we stratified plasma p-tau217 concentrations into tertiles and computed Spearman correlations in each tertile, allowing us to assess agreement at low, medium and high concentrations (Extended Data Fig. 2). Significant correlations were observed for p-tau217 concentrations in tertile two (*r*_S_ = 0.51; *P* < 0.0001) and tertile three (*r*_S_ = 0.62; *P* < 0.0001), but no relationship in tertile one, where all participants were CSF Aβ42/p-tau181 negative. The strength of the relationship between capillary and venous blood was not confounded by age or sex (Supplementary Table 2).

Next, we investigated the association of capillary biomarkers with cognitive testing. DPS p-tau217 showed significant correlations with both Mini Mental State Evaluation (MMSE) (*n* = 209; *r*_S_ = −0.374, 95% CI −0.485 to −0.251; *P* < 0.0001) (Fig. 2b) and age (*n* = 249; *r*_S_ = 0.334, 95% CI 0.219–0.440; *P* < 0.0001) (Fig. 2c), which were similar to the correlations of venous plasma p-tau217 with MMSE (*n* = 209; *r*_S_ = −0.410, 95% CI −0.517 to −0.290; *P* < 0.0001) (Fig. 2b) and age (*n* = 249; *r*_S_ = −0.317, 95% CI 0.200 to 0.424; *P* < 0.0001) (Fig. 2c).

### Diagnostic accuracy of capillary DPS p-tau217

**Fig. 3:**
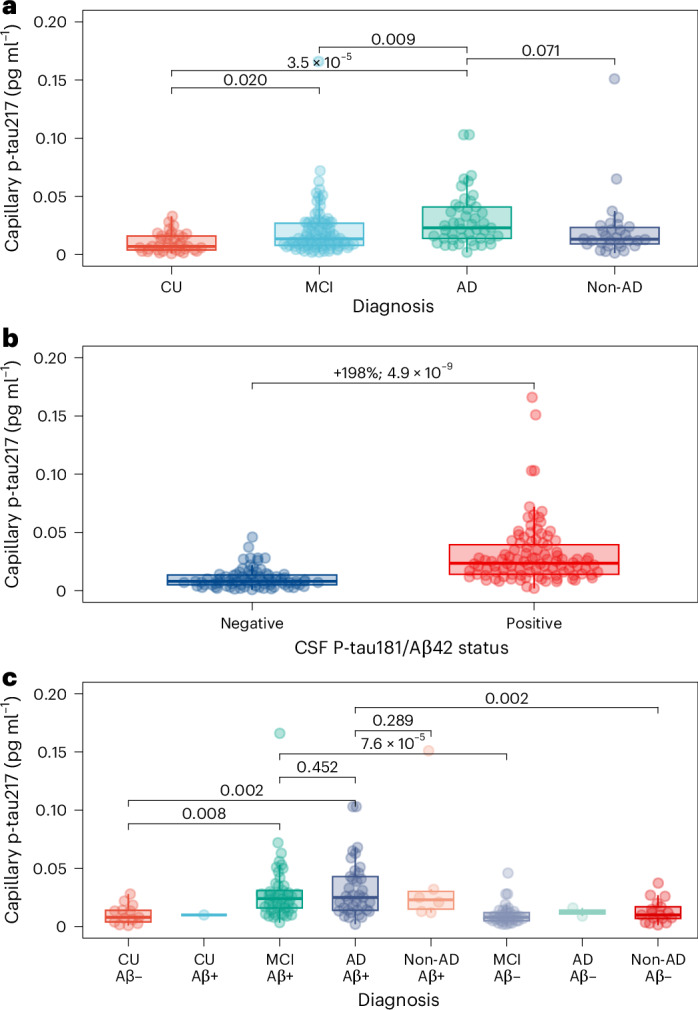
Capillary p-tau217 levels and their relationship to clinical diagnosis and CSF AD pathology status. **a**, Relationship between capillary p-tau217 level and diagnostic groups (CU, *n* = 37; MCI, *n* = 92; AD, *n* = 45; non-AD, *n* = 30). Horizontal solid-line bars represent group-wise comparisons alongside *P* values, obtained from post-hoc contrasting of a linear model adjusted for age and sex. **b**, Relationship between capillary p-tau217 level and CSF p-tau181/Aβ42 status (CSF-negative, n = 71; CSF-positive, n = 93). In addition to the *P* value, the mean fold-change between groups is presented. **c**, Relationship between capillary p-tau217 level and clinico-biological groups (CU Aβ−, *n* = 13; CU Aβ+, *n* = 1; MCI Aβ+, *n* = 49; AD Aβ+, *n* = 37; non-AD Aβ+, *n* = 6; MCI Aβ−, *n* = 39; AD Aβ−, *n* = 2; non-AD, *n* = 17), defined by clinical syndrome in conjunction with the CSF p-tau181/Aβ42 status, which is a validated metric for Aβ-positivity. In all panels, individual data points for each participant are shown and an overlaid boxplot represents group-wise distributions. Boxplots show the median (center line), interquartile range (IQR; box limits, 25th–75th percentiles), whiskers extending to the most extreme values within 1.5× IQR from the quartiles. A mean regression line is presented with ribbons representing 95% CI. Statistical tests were two-sided, and for group comparisons Tukey’s adjustment was used. Source data

**Extended Data Fig. 3:**
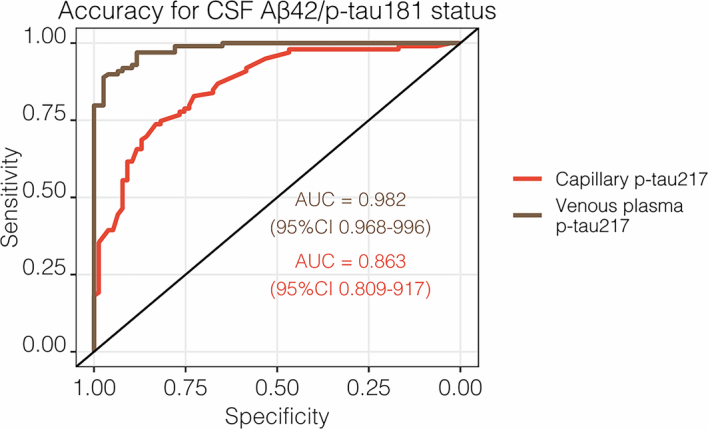
ROC curve for venous plasma vs capillary p-tau217 in discriminating CSF Aβ42/p-tau181 positivity. ROC curve representing the discriminative accuracies of capillary (red) and venous plasma (brown) p-tau217 for abnormal CSF Aβ42/p-tau181 values based on a dataset of individuals with paired venous plasma p-tau217, capillary p-tau217 and CSF data (n = 176). In their correspondent colors, AUCs and their 95% confidence intervals are displayed as text. Statistical tests were two-sided. Aβ = Amyloid-β; AUC = Area under the curve; CI = Confidence interval; CSF = Cerebrospinal fluid; p-tau = Phosphorylated tau; ROC = Receiver operating characteristic. Source data

**Extended Data Fig. 4:**
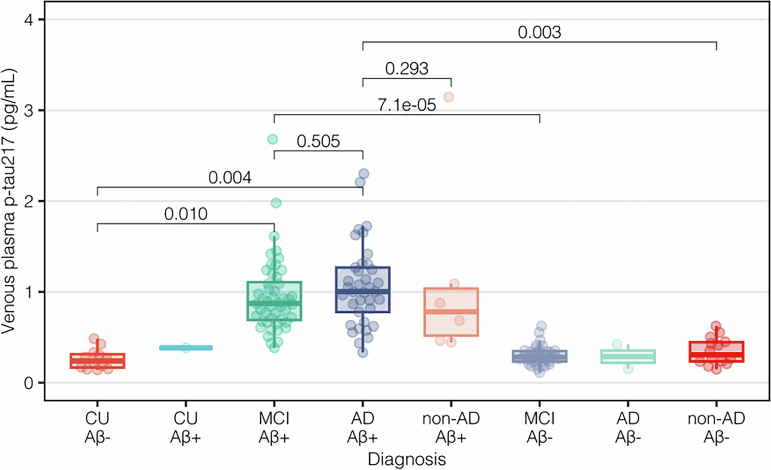
Venous plasma p-tau217 levels according to clinical-biological groups. Venous plasma p-tau217 levels (y-axis) are displayed with boxplots according to the clinical-biological groups, which combine clinical diagnosis with the CSF Aβ42/p-tau181 status of participants (CU Aβ–, n = 12; CU Aβ+, n = 1; MCI Aβ+, n = 48; AD Aβ+, n = 37; non-AD Aβ+, n = 6; MCI Aβ–, n = 39; AD Aβ–, n = 2; non-AD Aβ–, n = 14). P-values for between-group comparisons were obtained with post-hoc contrasts from linear models adjusted for age and sex and Tukey’s adjustment was used. Boxplots show the median (center line), interquartile range (box limits, 25th–75th percentiles), whiskers extending to the most extreme values within 1.5×IQR from the quartiles. Statistical tests were two-sided. Aβ = Amyloid-β; AD = Alzheimer’s disease; CSF = cerebrospinal fluid; CU = Cognitively unimpaired; IQR = Interquartile range; MCI = Mild cognitive impairment; p-tau = Phosphorylated tau. Source data

**Extended Data Fig. 5:**
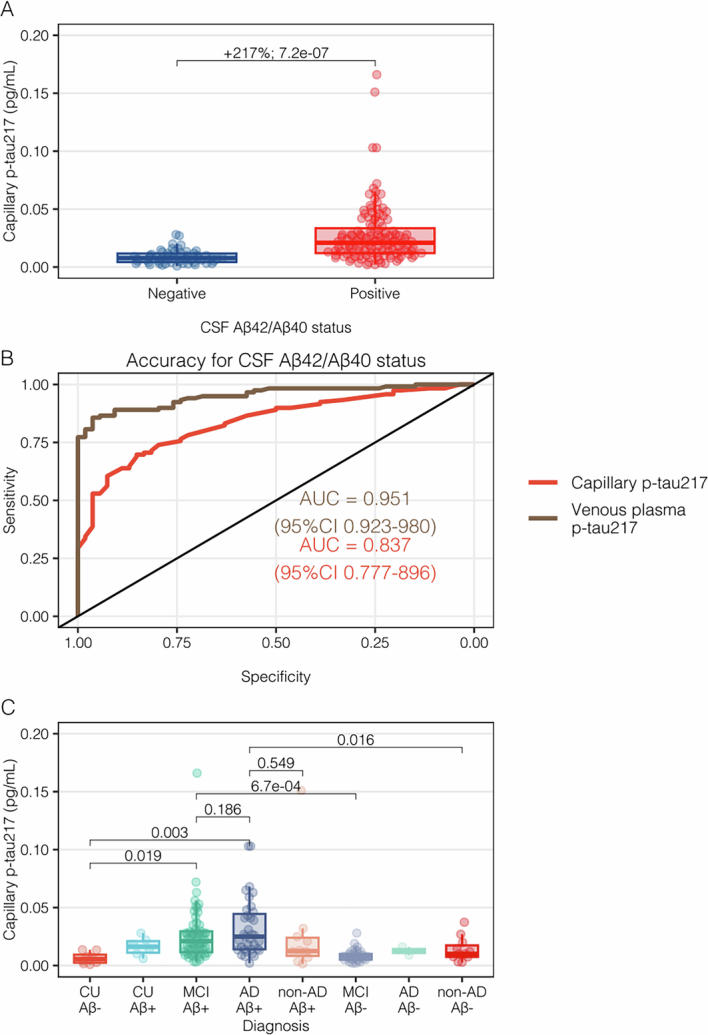
Group distribution and discrimination of capillary p-tau217 based on CSF Aβ42/Aβ40 as the standard of truth. (**A**) Boxplots indicating capillary p-tau217 concentrations (y-axis) according to CSF Aβ42/Aβ40 status (x-axis; blue for Aβ-negative, red for Aβ-positive; CSF-negative, n = 54; CSF-positive, n = 119). (**B**) ROC curve representing the discriminative accuracies of capillary (red) and venous plasma (brown) p-tau217 for abnormal CSF Aβ42/Aβ40 values (n = 173). In their correspondent colors, AUCs and their 95% confidence intervals are displayed as text. (**C**) Capillary p-tau217 levels (y-axis) are displayed with boxplots according to the clinical-biological groups, which combine clinical diagnosis with the CSF Aβ42/Aβ40 status of participants (CU Aβ–, n = 8; CU Aβ+, n = 6; MCI Aβ+, n = 59; AD Aβ+, n = 35; non-AD Aβ+, n = 10; MCI Aβ–, n = 29; AD Aβ–, n = 2; non-AD Aβ–, n = 11). P-values for between-group comparisons were obtained from group-contrasts from linear models adjusted for age and sex, with Tukey’s adjustment. Boxplots show the median (center line), interquartile range (box limits, 25th–75th percentiles), whiskers extending to the most extreme values within 1.5×IQR from the quartiles. Statistical tests were two-sided. Aβ = Amyloid-β; AD = Alzheimer’s disease; AUC = Area under the curve; CSF = Cerebrospinal fluid; CU = Cognitively unimpaired; IQR = Interquartile range; MCI = Mild cognitive impairment; p-tau217 = Phosphorylated tau 217; ROC = Receiver operating characteristic. Source data

**Extended Data Fig. 6:**
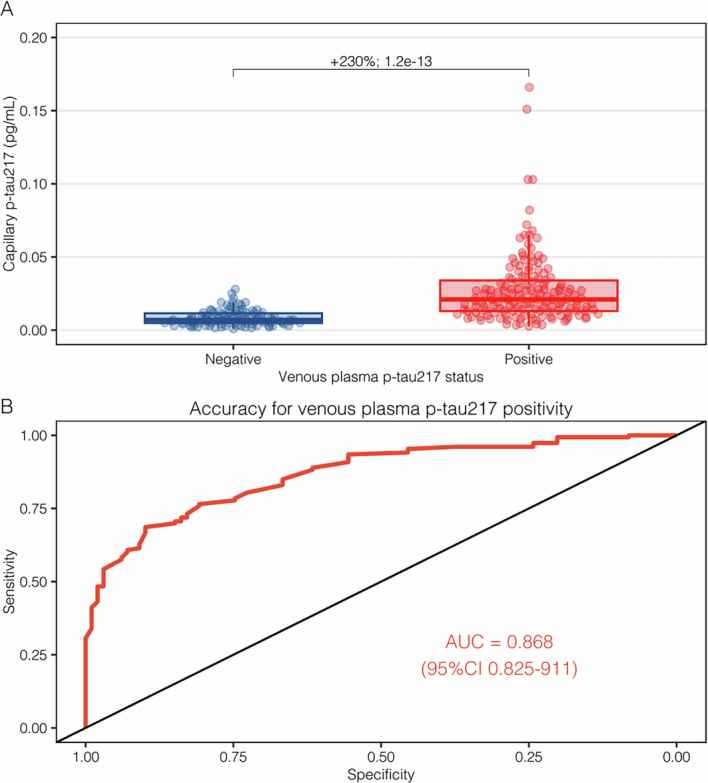
Capillary p-tau217 distribution and discrimination for plasma p-tau217 positivity. (**A**) Boxplots representing capillary p-tau217 concentrations (y-axis) according to venous plasma p-tau217 (x-axis; blue for venous plasma-negative [n = 99], red for venous plasma-positive [n = 153]) status. P-values for between-group comparisons were obtained from group-contrasts from linear models adjusted for age and sex, with Tukey’s adjustment. Boxplots show the median (center line), interquartile range (box limits, 25th–75th percentiles), whiskers extending to the most extreme values within 1.5×IQR from the quartiles. Statistical tests were two-sided. (**B**) ROC curve for the discriminative ability of capillary p-tau217 in detecting venous plasma p-tau217 positivity (n = 252). AUC = Area under the curve; IQR = Interquartile range; p-tau217 = Phosphorylated tau 217; ROC = Receiver operating characteristic. Source data

DPS p-tau217 was significantly increased in clinically defined mild cognitive impairment (MCI) and AD (no biomarker classification) compared with CU participants and clinically defined non-AD dementias (Fig. 3a). Next, we investigated the discriminative accuracy of DPS p-tau217 to detect abnormal CSF biomarkers. In participants with DPS p-tau217, venous plasma p-tau217 and CSF Aβ42/p-tau181 (*n* = 176; mean (s.d.) age, 74.6 (7.9) years; 102 women (58.0%)), capillary DPS p-tau217 was significantly increased (+198%; *P* < 0.001) in the AD CSF biomarker-positive group (Fig. 3b). DPS p-tau217 had an area under the curve (AUC) of 0.863 (95% CI 0.809–0.917); however, this was significantly lower than venous plasma p-tau217 which had an AUC of 0.982 (95% CI 0.968–0.996; *P* < 0.0001) (Extended Data Fig. 3) in the same participant subsample. We also show the distribution of capillary p-tau217 across clinico-biological diagnostic groups (Fig. 3c), which shows a similar pattern to venous derived p-tau217, with similar statistical significance across groups (Extended Data Fig. 4). Results demonstrating DPS p-tau217 against CSF Aβ42/Aβ40 as the standard of truth are shown in Extended Data Fig. 5. Next, we tested the accuracy of capillary DPS p-tau217 to determine abnormal venous plasma p-tau217 (*n* = 252), which had predetermined cutoff validated against Aβ-PET (ALZpath single molecule array (Simoa) > 0.42 pg ml^−1^)^3^. DPS p-tau217 was more concordant with venous plasma p-tau217 and was increased by 217% in individuals with venous plasma p-tau217 > 0.42 pg ml^−1^, compared with individuals with venous plasma p-tau217 ≤ 0.42 pg ml^−1^, and had a discriminative accuracy to detect abnormal venous plasma p-tau217 of 0.868 (95% CI 0.825–0.911) (Extended Data Fig. 6).

### Capillary DPS cutoffs based on CSF biomarker positivity

Exploratory diagnostic accuracy metrics were derived in a subset of individuals (*n* = 176) with paired capillary and CSF Aβ42/p-tau181 metrics, at a prevalence of 56.3% of CSF biomarker positivity. A capillary p-tau217 cutoff of 0.01 pg ml^−1^, with 90% sensitivity for abnormal CSF Aβ42/p-tau181, led to a positive predictive value (PPV) of 0.738 (95% CI 0.653–0.808) and a negative predictive value (NPV) of 0.833 (95% CI 0.713–0.910), at a specificity of 58.4%. A capillary cutoff of 0.02 pg ml^−1^, with 90% specificity for abnormal CSF Aβ42/p-tau181, led to a PPV of 0.884 (95% CI 0.789–0.940) and an NPV of 0.645 (95% CI 0.551–0.729), at a sensitivity of 66.7%. A Youden’s J statistic capillary cutoff (0.016 pg ml^−1^) led to a PPV of 0.849 (95% CI 0.758–0.909) and an NPV of 0.711 (95% CI 0.610–0.795), at a sensitivity of 73.7% and specificity of 83.2%. When combining the 90% sensitivity cutoff (0.01 pg ml^−1^) with the 90% specificity cutoff (0.02 pg ml^−1^) in a two-cutoff approach, the NPV of the lower cutoff was, as above, 0.833 (95% CI 0.713–0.910) and the PPV of the upper cutoff was 0.884 (95% CI 0.789–0.940), reaching an overall accuracy for those below the lower cutoff and above the upper cutoff of 86.2% (95% CI 78.8–91.7%), with 53 of the 176 individuals falling in the intermediate zone, which corresponded to 30.1% of the evaluated participants.

### GFAP and NfL in capillary DBS

**Fig. 4:**
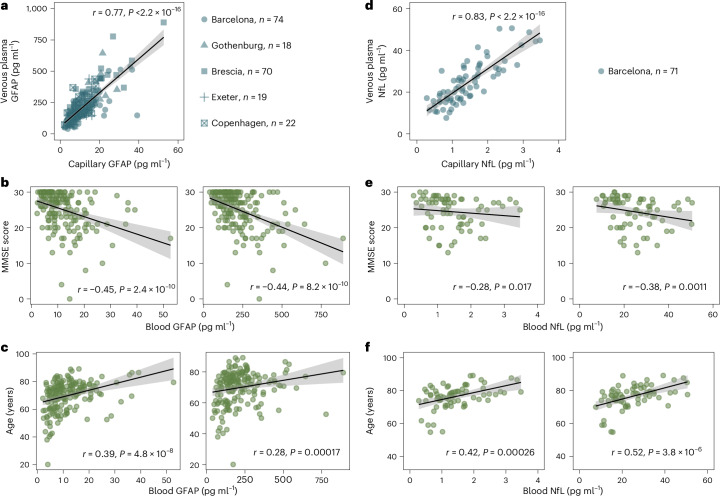
Associations between capillary GFAP and NfL with venous plasma assay, cognition and age. **a**, Association between venous plasma GFAP and capillary GFAP (*n* = 203). **b**,**c**, Association between MMSE score and blood GFAP (*n* = 181) (**b**), and between age and blood GFAP (*n* = 181) (**c**). Left: capillary GFAP levels. Right: venous plasma GFAP levels. **d**, Association between venous plasma NfL and capillary NfL for the Barcelona cohort (*n* = 71). **e**,**f**, Association between MMSE score and blood NfL (*n* = 71) (**e**), and between age and blood NfL (*n* = 71) (**f**). Left: capillary NfL levels. Right: venous plasma NfL levels. A mean regression line is presented in all panels, with ribbons representing 95% CI. For numerical representation of the correlation, we present Spearman coefficients alongside their *P* values. Statistical tests were two-sided, and for group comparisons Tukey’s adjustment was used. Source data

**Extended Data Fig. 7:**
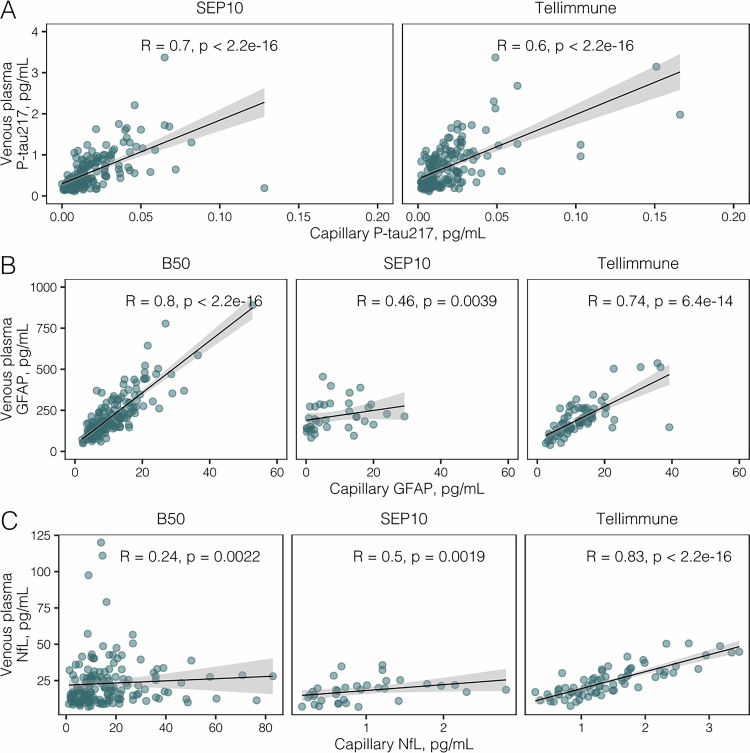
Comparative study of fingertip sampling method for capillary versus plasma biomarkers. Scatterplots showing the relationship between biomarkers measured in capillary blood (x-axis) and in venous plasma (y-axis) according to different capillary blood sampling methods (B50, SEP10, Telimmune) displayed in each panel. Row (**A**) indicates capillary and venous plasma relationships for p-tau217 (SEP10, n = 175; Telimmune, n = 173); row (**B**) for GFAP (B50, n = 162; SEP10, n = 37; Telimmune, n = 74); row (**C**) for NfL (B50, n = 162; SEP10, n = 36; Telimmune, n = 71). Spearman correlation coefficients are shown alongside their p-values and a mean regression line with 95% confidence intervals. Statistical tests were two-sided. GFAP = Glial fibrillary acidic protein; NfL = Neurofilament light; p-tau 217 = Phosphorylated tau 217. Source data

Based on 240 individuals (mean (s.d.) age, 69.9 (10.8) years; 99 women (48.8%)) measured using at least one of the three candidate DBS methods, we found that B50 and Telimmune collection cards were most compatible for GFAP and were combined for this analysis (Extended Data Fig. 7B). When comparing GFAP levels from capillary samples with venous plasma, a strong correlation was found (*r* = 0.773, 95% CI 0.710–0.823; *P* < 0.0001) (Fig. 4a). We also observed a similar correlation of capillary GFAP and venous plasma GFAP with age (capillary GFAP, *r* = 0.392, 95% CI 0.261–0.509; *P* < 0.0001; venous plasma GFAP, *r* = 0.276, 95% CI 0.135–0.405; *P* < 0.0001) (Fig. 4b) and MMSE (capillary GFAP, *r* = −0.448, 95% CI −0.558 to −0.324; *P* < 0.0001; venous plasma, *r* = −0.436, 95% CI −0.547 to −0.310; *P* < 0.0001) (Fig. 4c).

Based on a set with 237 individuals measured with at least one of the three DBS method candidates, only Telimmune DPS cards were useful in examining capillary NfL using our protocol (Extended Data Fig. 7C). Therefore, we examined 72 participants for NfL using Telimmune DPS cards (mean (s.d.) age, 76.4 (7.0) years; 45 women (62.5%)). When comparing NfL levels from capillary DPS to venous plasma, a strong correlation was observed (*r* = 0.83, 95% CI 0.743–0.892; *P* < 0.0001) (Fig. 4d). Similarly to GFAP, we observed similar correlations between DPS and venous plasma NfL in relation to age (capillary DPS, *r* = 0.429, 95% CI 0.219–0.601; *P* < 0.001; venous plasma, *r* = 0.524, 95% CI 0.333–0.674; *P* < 0.0001) (Fig. 4e) and MMSE (capillary DPS, *r* = −0.269, 95% CI −0.471 to −0.039; *P* = 0.02; venous plasma, *r* = −0.367, 95% CI −0.552 to −0.148; *P* < 0.001) (Fig. 4f).

### DPS p-tau217 and DBS GFAP in individuals with Down syndrome

**Fig. 5:**
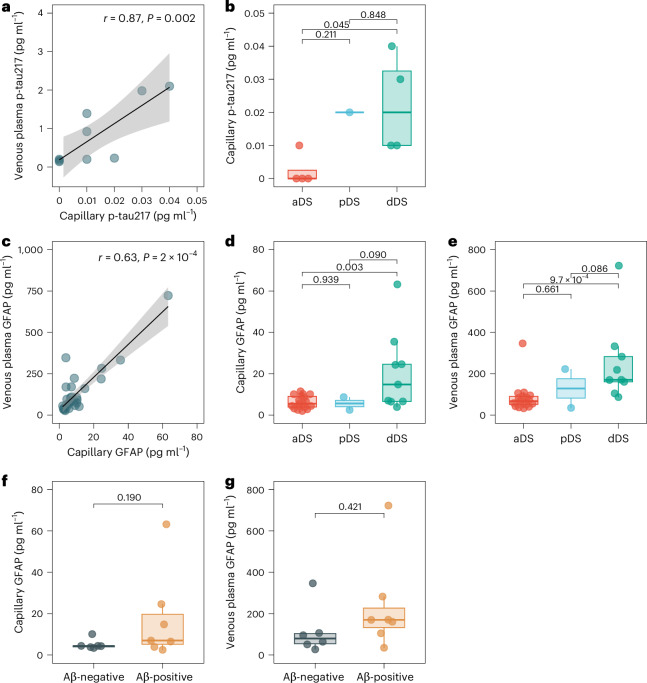
Associations between capillary p-tau217 and GFAP in participants with DS. **a**, Scatterplot representing the association between capillary and venous plasma p-tau217 in the DS Barcelona cohort, alongside their Spearman correlation coefficient and associated *P* value (*n* = 9). **b**, Boxplots of capillary p-tau217 based on cognitive diagnosis (aDS, *n* = 4; pDS, *n* = 1; dDS, *n* = 4). **c**, Scatterplot representing the association between capillary and venous plasma GFAP, with a Spearman correlation coefficient and its associated *P* value presented (*n* = 30). **d**, Boxplots of capillary GFAP based on cognitive diagnosis (aDS, *n* = 18; pDS, *n* = 2; dDS, *n* = 9). **e**, Boxplots of venous plasma GFAP based on cognitive diagnosis (aDS, *n* = 21; pDS, *n* = 3; dDS, *n* = 9). **f**,**g**, Boxplots of capillary GFAP (**f**) and venous plasma GFAP (**g**) based on CSF Aβ42/p-tau181 status (CSF-negative, *n* = 6; CSF-positive, *n* = 7). For scatterplots, a mean regression line is presented with 95% CI. All boxplots show the median (center line), IQR (box limits, 25th–75th percentiles), whiskers extending to the most extreme values within 1.5× IQR from the quartiles. When group comparisons are presented with boxplots, horizontal solid-line bars represent group-wise comparisons alongside *P* values, obtained from post-hoc contrasting of a linear model adjusted for age and sex. Statistical tests were two-sided, and for group comparisons Tukey adjustment was used. Source data

We examined 31 participants with DS and DBS biomarker data. As with the euploid participants, we found a significant relationship between biomarkers measured in capillary blood and venous blood (p-tau217, *r* = 0.875, 95% CI 0.503–0.973 (Fig. 5a) GFAP, *r* = 0.629, 95% CI 0.347–0.806 (Fig. 5c)). Capillary biomarker levels were, as also shown in venous plasma (Fig. 5e,f), increased in DS with dementia (dDS), compared with DS without AD-related cognitive impairment (aDS), for both p-tau217 (Fig. 5b) and GFAP (Fig. 5d). Participants positive for CSF p-tau181/Aβ42 more often had higher levels of capillary GFAP (Fig. 5f), although there were not sufficient participants with DS and DBS p-tau217 and CSF biomarker data (*n* = 5, CSF-negative only). Telimmune cards were not collected in this study, so no NfL results were obtained.

### Supervised and unsupervised capillary DPS or DBS collection

**Extended Data Fig. 8:**
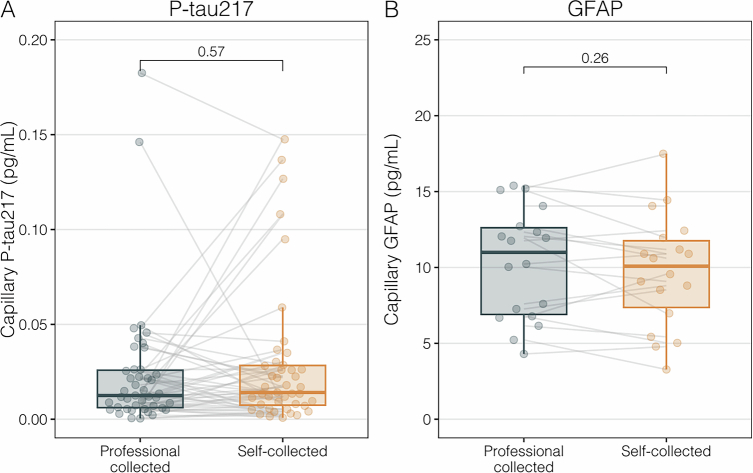
Supervised and unsupervised capillary DBS collection for p-tau217 and GFAP. Boxplots representing biomarker distributions for p-tau217 (**A**, n = 43) and GFAP (**B**, n = 18) in professionally collected dried blood spots versus self-collected, with p-value for between-group comparisons obtained from a non-parametric Wilcoxon signed rank test. Boxplots show the median (center line), interquartile range (box limits, 25th–75th percentiles), whiskers extending to the most extreme values within 1.5×IQR from the quartiles. GFAP = Glial fibrillary acidic protein; IQR = Interquartile range; p-tau217 = Phosphorylated tau 217. Source data

In the previous result sections, all capillary DPS or DBS collection was supervised and guided by trained personnel. Here we evaluated the within-person difference if collection was supervised compared with unsupervised. In 30 participants, capillary blood guided by study personnel and self-collected unsupervised samples showed a very high concordance with little difference between timepoints (DPS p-tau217, 0.014 pg ml^−1^ versus 0.013 pg ml^−1^, *P* = 0.57 (Extended Data Fig. 8A); DBS GFAP, 10.1 pg ml^−1^ versus 11.0 pg ml^−1^, *P* = 0.26 (Extended Data Fig. 8B)). Because only one Telimmune card was sampled per participant dedicated to p-tau217 quantification, no NfL data were obtained.

## Discussion

The DROP-AD project, constituting an effort to assess biomarkers for AD-type pathology and neurodegeneration from capillary blood, showcases the capability of quantifying p-tau217, GFAP and NfL protein levels. The study evaluated straightforward capillary blood collection methods, a new extraction protocol and ultrasensitive immunoassay biomarker determination. Biomarker levels from capillary blood correlated well with conventional venipuncture-collected plasma measures, and in the case of p-tau217, predicted with good accuracy, abnormal AD CSF biomarkers, as demonstrated in individuals classified as asymptomatic, MCI, dementia, as well as in individuals with DS, who are at high-risk for AD.

In blood, p-tau217 is the principal blood biomarker for determining AD pathology^8^ and is increasingly adopted as a reliable metric in research, clinical trials and clinical practice. It has the capabilities of high diagnostic accuracy to detect AD pathology, primarily amyloid^3,24^, but is also tightly associated with severity of tau pathology assessed by tangle counts at post-mortem examination^2^ and by tau PET during life^25^ not only in the symptomatic phase of the disease^4^, but also in the asymptomatic phase^26^. Therefore, p-tau217 holds promise not only for clinical use, but also population-level screening, identifying at-risk individuals in preclinical phases and enabling early intervention strategies^27^. Plasma p-tau217 has already been used to assess outcomes in secondary preventive trials^16,28^. A drawback in expanding blood biomarker testing outside specialized centers, is the strict protocol and guided venipuncture collection, sample handling and shipment. Dried blood sampling^23^ overcomes this limitation by enabling simplified, minimally invasive and potentially, remote self-collection, reducing the need for specialized personnel and facilitating broader population access to biomarker testing.

The DROP-AD project, conducted across multiple centers, highlights the strong potential of using dried capillary blood samples to accurately quantify plasma p-tau217. We observed robust correlations between p-tau217 concentrations measured from DPS and matched venous plasma samples, although the strength of these correlations varied by site. Importantly, p-tau217 levels showed a stepwise increase across clinical stages—CU, MCI and AD—and demonstrated good accuracy in predicting CSF biomarker-confirmed AD pathology. In addition to p-tau217, we successfully quantified GFAP and NfL using DBS and DPS matrices, respectively. Both GFAP and NfL showed high concordance between capillary and venous samples, and were similarly associated with cognitive performance and age, reinforcing the validity of these remote sampling methods. Although our primary focus was on biomarkers of AD neuropathology, the reliable detection of NfL from DPS samples has broader implications. Given its established role as a diagnostic, prognostic and monitoring biomarker, capillary-based NfL measurement could be transformative for other neurodegenerative and neurological conditions—including frontotemporal dementia, atypical parkinsonian syndromes, multiple sclerosis, amyotrophic lateral sclerosis and acute neurological injuries. Biomarker levels extracted from DPS or DBS cards, for all analytes of interest, were substantially lower than those quantified from venous plasma, which we believe is attributable to the elution of dried blood or plasma with buffer, resulting in dilution. Protein concentrations were not adjusted using a uniform dilution factor, because we cannot currently estimate the volume of plasma that is dried onto a card. Attempts to measure Aβ42 and Aβ40 using this technique yielded mixed results. Although Aβ40 was readily quantifiable, Aβ42 levels were mainly below the limit of detection and could not be included in the analysis, limiting the utility of this approach for this biomarker.

Imaging, CSF and blood-based biomarkers for AD pathology have shown strong translational applicability in individuals with DS, which represents the most common genetically determined form of AD^29^. Given the near-universal risk of AD in this population, there is a critical need for scalable and accessible methods to enable longitudinal biomarker monitoring, particularly in the context of preventive and disease-modifying clinical trials. Collection of blood samples by standard venipuncture may be complicated in individuals with DS—for example, due to relatively high rates of institutionalization and a lack of professionals—and remote blood collection thus offers a promising solution by reducing reliance on in-clinic visits and facilitating broader participation across diverse spectrum of intellectual disability. To evaluate the feasibility of this approach, we conducted a pilot study in which capillary blood samples were successfully collected from individuals with DS across a spectrum of cognitive stages. Our results revealed significantly elevated levels of capillary-derived GFAP and p-tau217 in participants with symptomatic AD compared with those who were cognitively asymptomatic. Importantly, biomarker concentrations derived from capillary samples showed strong concordance with those obtained from matched venous plasma, supporting the reliability and translational potential of remote sampling for biomarker quantification and ultimately, AD diagnosis, in this high-risk population.

This study has limitations. First, we have indicated that capillary blood collection may be useful in an unsupervised fashion, remotely. This has not been fully examined in this proof-of-principle study, where all capillary sampling was performed in research centers guided by trained staff. To gain some initial insights, we conducted a pilot in 30 participants who provided two capillary samples: one sampled by research staff and one unsupervised—1 h later. These initial findings demonstrate the reproducibility of both the collection method and the laboratory extraction procedure. Further, our venous plasma analyses were performed in single-batch analysis for all study sites, and this is particularly important to consider when comparing results directly to capillary testing, which was analyzed prospectively in multiple batches (less than 4 weeks from collection) throughout the 24-month study period. The observed lower accuracies to determine AD pathology by capillary p-tau217 could be partially attributed to this key difference in analytical design. This 24-month period also reflects a time of protocol optimization, in sample collection at multiple study sites and biomarker determination in the laboratory. Despite this optimization, we do experience a proportion of unsuccessful collections of capillary samples because of insufficient capillary blood flow, coagulation or technical issues during plasma separation in 15–25% of cases. This may, in part, reflect the inherent challenges of fingertip capillary blood sampling in clinical practice, where achieving consistent blood flow from a fingerstick collection is difficult and often complicated by hemolysis or admixture of interstitial fluid due to external compression of the fingertip^30^. We believe that diligent training of the study personnel and patients and/or caregivers and the provision of informational material is essential for successful collection of dried blood; however, alternative capillary blood collection methods—other than fingerstick—should be considered and examined given the encouraging finding from this study. Moreover, studies with larger cohorts are needed to investigate the impact of confounders on DPS or DBS biomarker levels.

In conclusion, our findings demonstrate that dried blood analysis offers a feasible and scalable approach for detecting AD pathology, particularly in research, population-based and epidemiological contexts. This minimally invasive method has the potential to substantially broaden our understanding of the prevalence and distribution of AD pathology across the general population, while also facilitating the inclusion of historically underrepresented populations and geographically diverse regions in AD research. However, despite the promise shown, we do not currently recommend the use of dried blood analysis for clinical use, decision-making or patient management, because of observed differences in analytical performance and diagnostic accuracy between capillary-derived and venous blood samples. Further methodological refinement and validation will be essential before clinical translation can be considered.

## Methods

### Study design

To evaluate the feasibility of capillary-derived blood as a simplified collection method compatible with AD biomarker analysis, paired venous plasma and capillary blood samples obtained by fingerstick were collected from CU and cognitively impaired individuals across seven European study centers. Capillary blood collection was conducted by trained study personnel at each site. Dried blood cards were shipped without temperature control to the Neurochemistry Laboratory at the University of Gothenburg, Sweden, within 1–40 days of collection. In parallel, venous plasma samples were stored at −80 °C at the respective study sites and shipped on dry ice to the same laboratory at the end of the study. Complementary CSF data (total *n* = 227; Barcelona, *n* = 131; Barcelona (DS), *n* = 13; Brescia, *n* = 7; Gothenburg, *n* = 13; Malmö, *n* = 40; Copenhagen, *n* = 23) and cognitive assessments (total *n* = 244; Barcelona, *n* = 130; Brescia, *n* = 32; Copenhagen, *n* = 24; Gothenburg, *n* = 14; Malmö, *n* = 44) were obtained from each site as part of routine clinical evaluations or existing research protocols.

### Cohort characteristics

At each study site, all participants provided written informed consent before enrollment, and the studies were approved by local ethical review authorities. The inclusion criteria for each cohort are depicted below and summarized in Supplementary Table 1. Participants were not compensated for participation in this study. Biological sex was determined based on self-identification.

The Ace Alzheimer Center Barcelona, Spain (the ‘Barcelona’ cohort) included participants under investigation for cognitive complaints recruited between September 2022 and April 2024. At Fundació ACE, clinical diagnosis was carried out through a comprehensive neuropsychological evaluation using the NBACE battery^31^, assessment of functional status with the Clinical Dementia Rating (CDR) scale, and supported by biological diagnosis through CSF biomarkers following the AT(N) classification framework^32^. Individuals with MCI and dementia were offered a voluntary (and informed consented) lumbar puncture in accordance with established consensus recommendations. Venous plasma, CSF and capillary DPS or DBS samples were collected on the same day under fasting conditions. All biospecimens obtained were part of the ACE collection, which was registered in Instituto de Salud Carlos III (ISCIII, Ministry of Health of Spain) under the code C.0000299. Capillary DPS and DBS samples were stored and shipped at room temperature between 1 and 3 days after the collection. The study was approved by the Ethics Committees of the Hospital Universitari de Bellvitge, Barcelona (Ref. PR148/22). The H70 Clinical Studies (the ‘Gothenburg’ cohort) consecutively recruited participants under investigation for cognitive symptoms from the memory clinic at the Sahlgrenska University Hospital in Gothenburg, Sweden between June 2023 and April 2024. There were no exclusion criteria. Capillary DPS and DBS samples, venous plasma and CSF samples were collected at the same study visit. Cognitive testing (MMSE and CDR) was performed in each participant. Capillary DPS and DBS samples were stored at room temperature and delivered to the Neurochemistry Laboratory between 1 and 7 days after the collection. Ethical approval for H70 Clinical Studies was provided by The Swedish Ethical Review Authority (Etikprövningsmyndigheten; EPM: 2023-06137-02). In the BioFINDER Primary Care (NCT06120361) and BioFINDER Preclinical AD (NCT06121544) studies (the ‘Malmö’ cohort), cognitively asymptomatic volunteers (asymptomatic AD or healthy controls) and individuals with cognitive symptoms undergoing cognitive diagnostic evaluation in primary care were included between December 2023 and November 2024. The exclusion criteria were (1) not undergoing CSF or blood sampling as part of clinical practice and (2) not undergoing cognitive testing as part of clinical practice. Cognitive testing (MMSE) and CSF samples were available for each participant. Capillary DPS and DBS were collected at the same day as venous plasma samples, stored at room temperature and shipped to the Neurochemistry Laboratory between 1 and 7 days after the collection. The studies were approved by Swedish Ethical Review Authority (Dnr. 2021-05724-01 and 2019-04320). Participants enrolled at the Center for Neurodegenerative Disorders at the University of Brescia, Italy (the ‘Brescia’ cohort) met current clinical criteria for the diagnosis of fontotemperal dementia^33,34^ or AD^35^, or were healthy individuals recruited among spouses or family members. Consecutive recruitment took place between October 2023 and June 2024. Each participant underwent an extensive clinical and neuropsychological evaluation and simultaneous venous EDTA plasma and dried blood spot collection. Cognitive testing (MMSE and CDR) was available for each participant and CSF samples were collected in a subgroup. Capillary DPS and DBS samples were stored at room temperature and shipped to the Neurochemistry Laboratory between 1 and 30 days after the collection. The study was approved by the local ethics committee (NP2189 and NP1965). Participants enrolled at the University of Exeter Medical School (the ‘Exeter’ cohort) were adults aged 50 years or above with a body mass index >25 kg m^−^^2^ and within 2 h travel of Exeter consecutively recruited from PROTECT-UK (Platform for Research Online to investigate Cognition and Genetics in Ageing) taking part in the DailyColors polyphenol supplement study in January 2024 (ref. ^36^). Exclusion criteria were the diagnosis of dementia and participation in an interventional clinical trial. Capillary dried blood samples and paired venous blood were collected at the same day. Capillary dried blood samples were stored at room temperature and shipped to the Neurochemistry Laboratory between 1 and 10 days after collection. This study was approved by the Ethics Committee of the University of Exeter, Faculty of Health & Life Sciences REC (Ref. 529634). Participants under investigation of a neurodegenerative disease from the memory clinic at Rigshospitalet, Copenhagen University Hospital (the ‘Copenhagen’ cohort) were enrolled between May 2024 and July 2024. For each participant, paired capillary DPS and DBS and venous plasma, sampled on the same day, and CSF and MMSE data were available. Individuals were excluded if they did not consent to the Danish Dementia Biobank, if the lumbar puncture was unsuccessful, or if they were clinically evaluated as incapable of participating in the project. Capillary dried blood samples were stored at room temperature and shipped within 7–40 days after collection. This study was approved by the Danish Research Ethics Committee (Ref. H-23078392). For the Sant Pau cohort, participants with DS with (dDS or prodromal AD (pDS)) and without AD-related cognitive impairment (aDS) were consecutively recruited at the Sant Pau Memory Unit, Barcelona, Spain from the Down Alzheimer Barcelona Neuroimaging Initiative (DABNI) study between May 2024 and November 2024. Capillary DPS and DBS samples were collected at the same day as venous EDTA plasma samples and stored at room temperature and shipped within 1–14 days after collection. This study was approved by the Sant Pau Ethics Committee. All participants or their legally authorized representative gave written informed consent before enrollment.

### Capillary blood collection and testing

Three different dried blood spot collection devices were used in this study. Capitainer SEP-10 (Capitainer AB), Capitainer B50 (Capitainer AB) and Telimmune Plasma Separation Card (Novilytic). Capitainer SEP-10 and the Telimmune Plasma Separation Card were used interchangeably to measure p-tau217. Capitainer B50 and the Telimmune Plasma Separation Card were utilized for GFAP quantification. Telimmune Plasma Separation Cards alone were used for NfL measurements based on comparative studies (Extended Data Fig. 7). In all cohorts, capillary blood was collected by study personnel from the middle or index finger using a single-use lancet with a 1.5-mm wide and 2.0-mm deep cut. In the Exeter cohort, a secondary unsupervised capillary blood collection was carried out by all participants on the same day.

The Capitainer B50 and Capitainer SEP-10 cards collect 50 μl and 70 μl of capillary whole blood, respectively. In the SEP-10 cards, blood cells are separated from the whole blood generating 10 μl of a plasma-like sample. Whole blood and plasma-like spots are left to dry at room temperature for 30 min. The Telimmune Plasma Separation Card does not restrict blood volume and 50 μl of capillary whole blood, equivalent to 6 μl of plasma-like sample, was pipetted from the finger to the card to standardize collection volume. Telimmune Plasma Separation Cards were left to dry for 3 min, then the cell separation membrane layer was removed, and the samples were left to dry for additional 30 min. After drying, all cards were stored at room temperature and shipped without temperature control or cooling on a regular basis to the Neurochemistry Laboratory, Gothenburg, Sweden. Before analysis, one filter disk from Capitainer B50 and Capitainer SEP-10 cards and both filter disks from the Telimmune Plasma Separation Cards were removed from the card using a semi-automated spot collector (Capitainer AB), and transferred to a deep 96-well plate (Sirocco protein precipitation plate, Waters). Samples were then incubated shaking with 170–300 μl of protein extraction buffer depending on the analyte of interest and at 37 °C and 500 rpm for 30 min; for p-tau217 quantification, filter papers were eluted with 170 μl buffer for all card types (Quanterix, catalogue number 105909); for N2PE and N4PE assays, Capitainer B50 filter papers were eluted with 300 μL and Telimmune filter papers with 170 μl of analyte-specific buffer (Quanterix, catalogue numbers 103659 (N4PE) and 103516 (N2PB)). After incubation, the samples were centrifuged at 20 °C and 2,626*g* for 15 min and the eluate was collected in a conical 96-well plate (Quanterix). After spinning, the filter disks and protein precipitation plate were discarded, and the eluate was immediately used for biomarker analysis in singlecates by Simoa technology on the HD-X platform using a neat protocol (ALZpath Simoa pTau-217 v2 Assay^3^ (Quanterix, catalogue number 104371), Simoa Neurology 4-plex E (Quanterix, catalogue number 103607) or Simoa Neurology 2-plex B (Quanterix, catalogue number 103520).

### Venous plasma and CSF biomarkers

Venous plasma samples collected by venipuncture and CSF collected by lumbar puncture are summarized for each cohort^37–42^. Before immunoassay procedures, venous EDTA plasma samples were thawed for 45 min at room temperature, vortexed for 30 s at 2,000 rpm and centrifuged at 4,000*g* and 20 °C for 10 min. Venous plasma samples were analyzed in singlecates with the same immunoassays using standard operating procedures, except for the Malmö cohort where venous plasma p-tau217 was quantified using an immunoassay on the Meso Scale Discovery platform developed by Lilly^2^. CSF biomarkers (Aβ42/Aβ40 or Aβ42/p-tau181) were analyzed by either Lumipulse G1200 (Fujirebio) or Elecsys e801 analyzers (Roche Diagnostics).

### Immunoassay quality control

For the Gothenburg cohort, paired capillary blood extracts and venous plasma samples were measured in the same experiment. In all other cohorts, for logistic reasons, capillary blood extracts were measured prospectively with the accompanying venous plasma samples measured in a single batch at the end of the study. High and low dried blood quality controls were developed for p-tau217, GFAP and NfL on Capitainer SEP-10 filter disks. Dried blood quality controls were extracted on the day of each experiment and measured in duplicates at the beginning and end of each plate. Moreover, additional high and low venous plasma controls were run in duplicates at the beginning and end of each plate. Here, the inter-assay coefficient of variation (CV) was <17% for DPS and <15% for venous plasma p-tau217; the intra-assay CV was <15% for DPS p-tau217 and <7% for venous plasma p-tau217; the inter-assay CV for NfL and GFAP was <22% for DPS and <15% for venous plasma; and the intra-assay CV for NfL and GFAP was <11% for DPS and <10% for venous plasma (Supplementary Table 3).

### Unsupervised capillary blood collection

To test the potential of capillary dried blood collection as self-sampling method, participants in the Exeter cohort underwent a second independently performed sampling session using Capitainer SEP-10, Capitainer B50 and Telimmune cards. During the first collection carried out by the study personnel, participants observed the sampling process and were handed written instructions (short text boxes and pictograms). Participants were then left alone with the instructions and performed the capillary dried blood collection independently (*n* = 44 pairs for DPS p-tau217; *n* = 18 pairs for DBS GFAP).

### Statistical analysis

Demographic information from participants were summarized with descriptive statistics, with mean and s.d. for continuous variables and counts and percentages for categorical variables. For visualizing associations between capillary dried blood spot biomarkers and variables of interest, we used scatterplots and presented a mean linear regression line to represent trends in associations. To numerically quantify and compare these associations, we used Spearman’s rho. To visualize between-group differences in DBS biomarkers, we plotted individual data points overlaid with boxplots. When group comparisons were made, we used linear models adjusted for age, sex and center (when applicable), and obtained *P* values from post-hoc Tukey contrasts between the levels of categorical variables. *P* values and 95% CIs were presented or described when appropriate. When evaluating biomarker discriminative ability for binary outcomes such as CSF biomarker positivity, we computed the AUC of receiver operating characteristics and used the DeLong test when receiver operating characteristic curves were compared. When evaluating diagnostic properties of DPS p-tau217, we assessed cutoffs derived with 90% sensitivity, 90% specificity or maximum Youdens’ Index for CSF or venous plasma biomarker positivity and computed their respective NPV and PPV based on the prevalence of biomarker positivity in the subset of participants with available data for each analysis. Statistical significance was set as a two-sided alpha = 0.05. We did not control for multiple comparisons, and statistical significance was interpreted taking this into consideration. All analyses were performed in R v.4.2.1 (2022-06-23) on macOS 15.6.1.

### Reporting summary

Further information on research design is available in the Nature Portfolio Reporting Summary linked to this article.

## Online content

Any methods, additional references, Nature Portfolio reporting summaries, source data, extended data, supplementary information, acknowledgements, peer review information; details of author contributions and competing interests; and statements of data and code availability are available at 10.1038/s41591-025-04080-0.

## Supplementary information

Supplementary Information Supplementary Tables 1–3.

Reporting Summary

## Source data

Source Data Figs. 1–5 and Extended Data Figs. 1–8 Statistical source data Figs. 1–5 and Extended Data Figs. 1–8.

## Data Availability

This study includes no data deposited in external repositories. Blinded and anonymized data can be shared with academic investigators, for the sole purpose of replicating procedures and results presented in the article, as long as data transfer agrees with local legislation and with the local Ethical Review Board of each cohort, which must be regulated in a material or data transfer agreement. Researchers interested in accessing the datasets should contact the corresponding author (Nicholas.Ashton@Bannerhealth.com) and provide a brief research proposal outlining the intended use of the data. Data requests will be evaluated based on scientific merit and compliance with ethical and legal requirements; requests are typically processed and accepted within 2–3 months. Data displayed in this paper have been provided in the source data file. Source data are provided with this paper.

## Code availability

All analyses were performed in R v.4.2.1 (2022-06-23) on macOS 15.6.1. The R code that supports the main results of this study is publicly available on GitHub (https://github.com/wsbrum/dropad_natmed). All models were built using publicly available packages and functions in the R programming language.
