# Supplementary material for: A minimally invasive dried blood spot biomarker test for the detection of Alzheimer’s disease pathology
Source: Nat Med. 2026 Jan 5;32(2):599–608. doi: 10.1038/s41591-025-04080-0 (PMC12920126; doi:10.1038/s41591-025-04080-0)
Supplement: Supplementary file 1 — Supplementary Tables 1–3. [file 41591_2025_4080_MOESM1_ESM.pdf]

nature medicine

Supplementary information https://doi.org/10.1038/s41591-025-04080-0

A minimally invasive dried blood spot biomarker test for the detection of Alzheimer's disease pathology

In the format provided by the authors and unedited

Table of Contents

Page 1 – **Supplementary Table 1**. Cohort characteristics

Page 2 – **Supplementary Table 2**. Multivariable regression models of capillary p-tau217 with plasma p-tau217 and covariates

Page 3 – **Supplementary Table 3**. Assay repeatability and intermediate precision

Supplementary Table 1. Cohorts characteristics

**Study side**	**Inclusion criteria**	**Exclusion criteria**	**Sampling period**	**Shipping range; temperature**	**Cognitive testing available**	**CSF data available**	**Ethical approval**
**The "Barcelona cohort"** Ace Alzheimer Center Barcelona, Spain	Participants under investigation for cognitive complaints	None	09/2022 - 04/2024	1-3 days after collection; RT	Yes (CDR)	Yes (sub group)	Ethics Committees of the Hospital Universitari de Bellvitge, Barcelona (Ref. PR148/22)
**The "Gothenburg cohort"** Memory clinic, Sahlgrenska University Hospital Gothenburg, Sweden	Participants under investigation for cognitive complaints	None	06/2023 - 04/2024	1-7 days after collection; RT	Yes (MMSE and CDR)	Yes	The Swedish Ethical Review Authority (Etikprövningsmyndigheten; EPM: 2023-06137-02)
**The "Malmö cohort"** *BioFINDER Primary Care* and BioFINDER Preclinical AD studies Malmö, Sweden	Asymptomatic AD, healthy controls, patients with cognitive symptoms undergoing diagnostic evaluation in primary care	No CSF/ blood sampling/ cognitive testing as part of clinical practice	12/2023 - 11/2024	1-7 days after collection; RT	Yes (MMSE)	Yes	Swedish Ethical Review Authority (Dnr. 2021-05724-01 and 2019-04320)
**The "Brescia cohort"** Center for Neurodegenerative Disorders, University of Brescia, Italy	Individuals with FTD or AD, and healthy spouses/ family members	None	10/2024 - 06/2024	1-30 days after collection; RT	Yes (MMSE and CDR)	Yes (sub group)	Local Ethics Committee University of Bresica (NP2189 and NP1965)
**The "Exeter cohort"** University of Exeter Medical School, United Kingdome	Participants taking part in the DailyColors polyphenol supplement study: ≥ 50 years of age, BMI ≥ 25 kg/m²	Dementia diagnosis; participation in a clinical trial; diet-related factors described previously	01/2024	1-10 days after collection; RT	No	No	Ethics Committee of the University of Exeter, Faculty of Health & Life Sciences REC (Ref. 529634)
**The "Copenhagen" cohort** Memory clinic, Rigshospitalet, Copenhagen University Hospital, Denmark	Participants under investigation of a neurodegenerative disease	No consent to the Danish Dementia Biobank; no lumbar puncture; clinically incapability to participate	05/2024 - 07/2024	7-40 days after collection; RT	Yes (MMSE)	Yes	Danish Research Ethics Committee (Ref. H-23078392)
**The "Sant Pau cohort"** Sant Pau Memory Unit, Barcelona, Spain	Participants with DS with AD or without AD-related cognitive impairment taking part in the DABNI study	None	05/2024 - 11/2024	1-14 days after collection; RT	No	Yes (sub group)	Sant Pau Ethics Committee

AD = Alzheimer's disease; BMI = Body mass index; CDR = Clinical dementia rating; CSF = Cerebrospinal fluid; DABNI = Down Alzheimer Barcelona Neuroimaging Initiativ; DS = Down Syndrome; FTD = Frontotemporal dementia; MMSE = Minimental state examination; RT = Room temperature

Supplementary Table 2. Multivariable regression models of capillary p-tau217 with plasma p-tau217 and covariates

	**Model 1: Plasma only**		**Model 2: + Demographics**		**Model 3: + Diagnosis**		**Model 4: + CSF status**	
Predictors	*Std. Beta*	*p*	*Std. Beta*	*p*	*Std. Beta*	*p*	*Std. Beta*	*p*
(Intercept)	0.00	0.975	0.01	0.611	0.13	0.576	0.19	0.460
Plasma p-tau217	0.69	**<0.001**	0.68	**<0.001**	0.70	**<0.001**	0.75	**<0.001**
Age			0.03	0.589	0.05	0.451	0.06	0.354
Sex (Female)			-0.01	0.908	0.01	0.953	0.00	0.983
Diagnosis (MCI)					-0.15	0.515	-0.11	0.631
Diagnosis (AD)					-0.24	0.366	-0.18	0.511
Diagnosis (Non-AD)					-0.01	0.979	-0.00	0.990
CSF (Pos)							-0.15	0.395
Observations	159		159		159		159	
R^2 / R^2 adjusted	0.475 / 0.471		0.476 / 0.465		0.481 / 0.460		0.483 / 0.459	

Standardized β-estimates and p-values from regression models predicting capillary p-tau217 levels. Model 1 includes plasma p-tau217 only; Model 2 additionally adjusts for age and sex; Model 3 additionally adjusts for diagnosis (MCI, AD, non-AD); Model 4 additionally adjusts for CSF Aβ42/P-tau181 status. Plasma p-tau217 remained the only significant predictor across all models. Aβ = Amyloid beta: AD = Alzheimer's disease; CSF = Cerebrospinal fluid; MCI = Mild cognitive impairment; p-tau217 = Phosphorylated tau 217; R^2 = Coefficient of determination; Std. Beta = Standardized beta coefficient

Supplementary Table 3. Assay repeatability and intermediate precision.

	DPS iQCS				Plasma iQCs			
	ID	Mean (pg/mL)	Intra-assay CV (%)	Inter-assay CV (%)	ID	Mean (pg/mL)	Intra-assay CV (%)	Inter-assay CV (%)
p-tau217 low	iQC1	0.022	7.3	13.1	iQC1<br>iQC2<br>iQC3<br>iQC4	0.6<br>0.4<br>0.4<br>0.1	5.7<br>5.2<br>4<br>6.8	7.7<br>10.3<br>12.8<br>7.2
p-tau217 high	iQC1	0.087	14.8	17.3	iQC1<br>iQC2<br>iQC3	2.0<br>1.8<br>0.6	2.8<br>5.3<br>2.3	7.5<br>11.8<br>14.1
GFAP low	iQC1	11.6	7.7	22.2	iQC1<br>iQC2<br>iQC3	96.2<br>82.7<br>119.3	6.0<br>5.7<br>7.8	11.5<br>9.7<br>7.8
GFAP high	iQC1	64.9	3.0	16.8	iQC1<br>iQC2<br>iQC3<br>iQC4	279.5<br>680.8<br>378.1<br>630.2	5.8<br>4.3<br>5.6<br>5.0	8.8<br>11.2<br>11.0<br>5.0
NfL low	iQC1	2.7	10.7	11.9	iQC1<br>iQC2<br>iQC3	16.7<br>14.6<br>15.8	5.8<br>4.3<br>9.7	10.0<br>7.5<br>9.7
NfL high	iQC1	11.3	8.4	13.6	iQC1<br>iQC2<br>iQC3<br>iQC4	87.5<br>96.3<br>184.0<br>92.5	5.9<br>5.6<br>6.1<br>6.8	9.3<br>7.9<br>14.5<br>7.9

For each analyte, two plasma and DPS iQC levels (high and low) were run in duplicates in the beginning and end of each plate. Reported is the analyte-specific mean of low and high iQC samples and the respective intra- and assay-CV. During the evolution of the DROP-AD project, up to four different iQC levels have been used for quality control purposes. CV = coefficient of variance; DPS = Dried plasma spots; GFAP = Glial fibrillary acidic protein; iQC = In-house quality controls; NfL = Neurofilament light; p-tau217 = Phosphorylated tau 217
