# Supplementary material for: A minimally invasive dried blood spot biomarker test for the detection of Alzheimer’s disease pathology
Source: Nat Med. 2026 Jan 5;32(2):599–608. doi: 10.1038/s41591-025-04080-0 (PMC12920126; doi:10.1038/s41591-025-04080-0)
Supplement: Supplementary file 2 — Reporting Summary [file 41591_2025_4080_MOESM2_ESM.pdf]

Corresponding author(s): N. J. Ashton

Last updated by author(s): Oct 16, 2025

Reporting Summary

Nature Portfolio wishes to improve the reproducibility of the work that we publish. This form provides structure for consistency and transparency in reporting. For further information on Nature Portfolio policies, see our Editorial Policies and the Editorial Policy Checklist.

Statistics

For all statistical analyses, confirm that the following items are present in the figure legend, table legend, main text, or Methods section.

n/a	Confirmed	
☐	☒	The exact sample size (*n*) for each experimental group/condition, given as a discrete number and unit of measurement
☐	☒	A statement on whether measurements were taken from distinct samples or whether the same sample was measured repeatedly
☐	☒	The statistical test(s) used AND whether they are one- or two-sided<br>*Only common tests should be described solely by name; describe more complex techniques in the Methods section.*
☐	☒	A description of all covariates tested
☐	☒	A description of any assumptions or corrections, such as tests of normality and adjustment for multiple comparisons
☐	☒	A full description of the statistical parameters including central tendency (e.g. means) or other basic estimates (e.g. regression coefficient) AND variation (e.g. standard deviation) or associated estimates of uncertainty (e.g. confidence intervals)
☐	☒	For null hypothesis testing, the test statistic (e.g. *F*, *t*, *r*) with confidence intervals, effect sizes, degrees of freedom and *P* value noted<br>*Give P values as exact values whenever suitable.*
☒	☐	For Bayesian analysis, information on the choice of priors and Markov chain Monte Carlo settings
☒	☐	For hierarchical and complex designs, identification of the appropriate level for tests and full reporting of outcomes
☐	☒	Estimates of effect sizes (e.g. Cohen's *d*, Pearson's *r*), indicating how they were calculated

Our web collection on statistics for biologists contains articles on many of the points above.

Software and code

Policy information about availability of computer code

Data collection	No software was used for data collection.
Data analysis	All analyses were performed in R version 4.2.1 (2022-06-23) on macOS 15.6.1. All analyses were performed in R version 4.2.1 (2022-06-23) on macOS 15.6.1. The R code that supports the main results of this study is publicly available on GitHub (https://github.com/wsbrum/dropad_natmed). All models were built using publicly available packages and functions in the R programming language. The key R packages used were ggplot2 (3.5.1), dplyr (1.1.4), tidyr (1.3.0), ggpubr (0.4.0), ggsignif (0.6.4), ggbeeswarm (0.6.0), ggh4x (0.2.2), ggsci (2.9), emmeans (1.8.4-1), Cairo (1.6-2), scales (1.3.0), pROC (1.18.0), and OptimalCutpoints (1.1-5).

For manuscripts utilizing custom algorithms or software that are central to the research but not yet described in published literature, software must be made available to editors and reviewers. We strongly encourage code deposition in a community repository (e.g. GitHub). See the Nature Portfolio guidelines for submitting code & software for further information.

Data

Policy information about availability of data

All manuscripts must include a data availability statement. This statement should provide the following information, where applicable:

- Accession codes, unique identifiers, or web links for publicly available datasets
- A description of any restrictions on data availability
- For clinical datasets or third party data, please ensure that the statement adheres to our policy

This study includes no data deposited in external repositories. Blinded and anonymized data can be shared with academic investigators, for the sole purpose of replicating procedures and results presented in the article, as long as data transfer agrees with local legislation and with the local Ethical Review Board of each cohort, which must be regulated in a material/data transfer agreement. Researchers interested in accessing the datasets should contact the corresponding author (Nicholas.Ashton@Bannerhealth.com) and provide a brief research proposal outlining the intended use of the data. Data requests will be evaluated based on scientific merit and compliance with ethical and legal requirements; requests are typically processed and accepted within 2-3 months. Data displayed in this manuscript have been provided in the source data file.

Research involving human participants, their data, or biological material

Policy information about studies with human participants or human data. See also policy information about sex, gender (identity/presentation), and sexual orientation and race, ethnicity and racism.

Reporting on sex and gender	Biological sex was determined based on self-identification; the term sex (biological attribute) was used in the manuscript. The study includes both men and women. In all 7 independent cohorts, 53.4% were female. Due to the balance of both sexes, we believe that the findings apply to both sexes.
Reporting on race, ethnicity, or other socially relevant groupings	This study does not include any reference to race, ethnicity, or other socially relevant groupings.
Population characteristics	In this study, we recruited 337 participants (mean [SD] age, 70.8 [11.7] years; 167 females [53.4%]) from seven centres in Sweden, Spain, Italy, United Kingdom and Denmark who were cognitively healthy, or had MCI or AD dementia. Detailed information is given in Table 1.
Recruitment	All participants at all seven study sides were recruited as part of undergoing a memory investigation or healthy controls.<br><br>Barcelona cohort: Participants under investigation for cognitive complaints were recruited between September 2022 and April 2024 at the Ace Alzheimer Center Barcelona, Spain. There were no exclusion criteria.<br><br>Gothenburg cohort: Participants under investigation for cognitive complaints were recruited between June 2023 and April 2024 at the Memory clinic at the Sahlgrenska University Hospital in Gothenburg, Sweden. There are no exclusion criteria.<br><br>Malmö cohort: Cognitively asymptomatic volunteers (asymptomatic AD or healthy controls) and patients with cognitive symptoms undergoing cognitive diagnostic evaluation in primary care from the BioFINDER Primary Care (NCT06120361) and BioFINDER Preclinical AD (NCT06121544) studies were recruited between December 2023 and November 2024 in Sweden. Exclusion criteria: 1) not undergoing CSF or blood sampling as part of clinical practice and 2) not undergoing cognitive testing as part of clinical practice.<br><br>Brescia cohort: Participants who met current clinical criteria for the diagnosis of FTD or AD, or healthy individuals recruited among spouses or family members were enrolled at the Center for Neurodegenerative Disorders at the University of Brescia, Italy between October 2023 and June 2024. There were no exclusion criteria.<br><br>Exeter cohort: Adults aged 50 years or above with a body mass index >25 kg/m² and within 2 hours travel of Exeter were consecutively recruited from PROTECT-UK (Platform for Research Online to investigate Cognition and Genetics in Ageing) in January 2024 at the University of Exeter Medical School, United Kingdom. Exclusion criteria: Diagnosis of dementia and participation in an interventional clinical trial.<br><br>Copenhagen cohort: Participants under investigation for neurodegenerative diseases were recruited from the Memory Clinic at Ringshospitalet, Copenhagen University, Denmark between May 2024 and July 2024. Exclusion criteria: not consenting to data and sample storage in the Danish Dementia Biobank, not undergoing a lumbar puncture during their clinical evaluation, or if they were clinically evaluated as incapable of participating in the project.<br><br>Sant Pau cohort (Barcelona DS cohort): Participants with Down Syndrome (DS) with and without AD related cognitive impairment were consecutively recruited at the Sant Pau Memory Unit, Barcelona, Spain from the Down Alzheimer Barcelona Neuroimaging Initiative (DABNI) study between May 2024 and November 2024. There were no exclusion criteria.
Ethics oversight	Gothenburg cohort: The Swedish Ethical Review Authority (Etikprövningsmyndigheten; EPM: 2023-06137-02)<br>Malmö cohort: Swedish Ethical Review Authority (Dnr. 2021-05724-01 and 2019-04320)<br>Brescia cohort: Local Ethics Committee at the University of Brescia (NP2189 and NP1965)<br>Exeter cohort: Ethics Committee of the University of Exeter, Faculty of Health & Life Science REC (Ref. 529634)<br>Copenhagen cohort: Danish Research Ethics Committee (Ref. H-23078392)<br>Sant Pau cohort: Sant Pau Ethics Committee

Note that full information on the approval of the study protocol must also be provided in the manuscript.

Field-specific reporting

Please select the one below that is the best fit for your research. If you are not sure, read the appropriate sections before making your selection.

☒ Life sciences ☐ Behavioural & social sciences ☐ Ecological, evolutionary & environmental sciences

For a reference copy of the document with all sections, see nature.com/documents/nr-reporting-summary-flat.pdf

Life sciences study design

All studies must disclose on these points even when the disclosure is negative.

Sample size	This study included a large samples size (n=337) across 7 different European centers. Due to the prospective, continuous sample collection, no sample size calculation has been performed.
Data exclusions	No data has been excluded from the analyses.
Replication	This was a multi-center study using 7 independent cohorts to replicate the findings. The results have been presented separately for each study side, emphasizing that we found comparable results across independent cohorts.
Randomization	This is an observational study and no allocation into experimental groups has been performed. Therefore, randomization is not relevant for this study.
Blinding	Biomarker analysis was performed by scientists blinded to the cognitive status/ diagnosis and participant information.

Reporting for specific materials, systems and methods

We require information from authors about some types of materials, experimental systems and methods used in many studies. Here, indicate whether each material, system or method listed is relevant to your study. If you are not sure if a list item applies to your research, read the appropriate section before selecting a response.

Materials & experimental systems

n/a	Involved in the study	
☐	☒	Antibodies
☒	☐	Eukaryotic cell lines
☒	☐	Palaeontology and archaeology
☒	☐	Animals and other organisms
☐	☒	Clinical data
☒	☐	Dual use research of concern
☒	☐	Plants

Methods

n/a	Involved in the study	
☒	☐	ChIP-seq
☒	☐	Flow cytometry
☒	☐	MRI-based neuroimaging

Antibodies

Antibodies used	GFAP, NFL and p-tau217 capture and detection antibodies provided with a commercially available immunoassay kit (Quanterix, Billerica, US)
Validation	For biomarker determination, commercially available immuno assays have been used (Quanterix, Billerica, US) and antibody selection is propriety, but they have widely been reported in academic publications (e.g., Palmqvist et al (jama.2020.12134), Thijssen et al (alz.038179))

Clinical data

Policy information about clinical studies

All manuscripts should comply with the ICMJE guidelines for publication of clinical research and a completed CONSORT checklist must be included with all submissions.

Clinical trial registration	Barcelona cohort: Instituto de Salud Carlos III Reg. biobank registry (C.0000299), Malmö cohort: BioFINDER Primary Care (NCT06120361), BioFINDER Preclinical AD (NCT06121544)
Study protocol	Barcelona cohort: https://www.fundacioace.com/en/clinical-research.html<br>Gothenburg cohort: https://www.gu.se/en/research/clinical-dementia-research-h70-clinical-studies<br>Malmö cohort: https://biofinder.se/memory-clinic/ and https://biofinder.se/primare-care/<br>Brescia cohort: DOI: 10.1002/alz.70289

Exeter cohort: https://www.protectstudy.org.uk/
Copenhagen cohort: s13195-024-01658-7
Sant Pau cohort: jalz.2016.06.713

Data collection

Barcelona cohort: Data collection at the Ace Alzheimer Center Barcelona, Spain, between September 2022 and April 2024.

Gothenburg cohort: Data collection at the Memory clinic at the Sahlgrenska University Hospital in Gothenburg, Sweden, between June 2023 and April 2024 .

Malmö cohort: Data collection from the BioFINDER Primary Care (NCT06120361) and BioFINDER Preclinical AD (NCT06121544) studies in Sweden between December 2023 and November 2024.

Brescia cohort: Data collection at the Center for Neurodegenerative Disorders at the University of Brescia, Italy between October 2023 and June 2024.

Exeter cohort: Data collection at the University of Exeter Medical School, United Kingdom in January 2024.

Copenhagen cohort: Data collection at the Memory Clinic at Ringshospitalet, Copenhagen University, Denmark between May 2024 and July 2024.

Sant Pau cohort: Data collection at the Sant Pau Memory Unit, Barcelona, Spain between May 2024 and November 2024.

Outcomes

The predefined primary outcomes of this analysis were p-tau217, NfL and GFAP levels from capillary dried blood spots.

Plants

Seed stocks

Report on the source of all seed stocks or other plant material used. If applicable, state the seed stock centre and catalogue number. If plant specimens were collected from the field, describe the collection location, date and sampling procedures.

Novel plant genotypes

Describe the methods by which all novel plant genotypes were produced. This includes those generated by transgenic approaches, gene editing, chemical/radiation-based mutagenesis and hybridization. For transgenic lines, describe the transformation method, the number of independent lines analyzed and the generation upon which experiments were performed. For gene-edited lines, describe the editor used, the endogenous sequence targeted for editing, the targeting guide RNA sequence (if applicable) and how the editor was applied.

Authentication

Describe any authentication procedures for each seed stock used or novel genotype generated. Describe any experiments used to assess the effect of a mutation and, where applicable, how potential secondary effects (e.g. second site T-DNA insertions, mosiacism, off-target gene editing) were examined.
